# Qualitative Analysis of Test-to-Treat Benefits and Barriers for Pharmacists in Rural Washington State

**DOI:** 10.3390/pharmacy13030080

**Published:** 2025-06-03

**Authors:** Bradley Brown, Megan Undeberg, Angela Stewart, Kimberly McKeirnan

**Affiliations:** 1 College of Pharmacy and Pharmaceutical Sciences, Washington State University, Yakima Campus, Yakima, WA 98901, USA; bradley.m.brown@wsu.edu (B.B.); stewas@wsu.edu (A.S.); 2 College of Pharmacy and Pharmaceutical Sciences, Washington State University, Spokane Campus, Spokane, WA 99202, USA; meganru@wsu.edu

**Keywords:** rural health, access to healthcare, community pharmacy, point-of-care testing, pharmacy practice

## Abstract

Background: Rural communities in the United States experience significant barriers in accessing healthcare, including inadequate numbers of providers and local healthcare facilities. These barriers are exacerbated during seasons with high rates of respiratory diseases when rural clinics and providers may be overwhelmed. When mild, many of these respiratory diseases may be managed effectively in alternate settings, including community pharmacies. Investigators interviewed pharmacists in Washington State to explore the capacity of pharmacists and pharmacies to provide test-to-treat services for COVID-19, influenza, and strep throat. Methods: A qualitative study design was used to conduct key informant interviews with pharmacists who precepted student pharmacists from a local university. Twenty interviews were conducted, transcribed, and qualitatively evaluated to identify themes. The 5 A’s of Access were utilized as a theoretical framework. This framework describes five domains of access, including affordability, availability, accessibility, accommodation, and acceptability. Results: Qualitative analysis identified several themes that described the benefits of offering test-to-treat services in rural communities, such as reducing geographical barriers to accessing care, reducing wait times for patients, and reducing the number of patients seeking higher levels of care for basic treatments. Barriers to offering test-to-treat services identified by pharmacist participants included difficulties with receiving payment for services, challenges with adequate staffing, and the lack of awareness among many people in rural communities that pharmacies offer test-to-treat services. Conclusions: Rural communities experience challenges with the limited capacity of healthcare providers to meet the needs of patients in their communities. The results of this qualitative analysis may be useful to pharmacists in U.S. states where collaborative drug therapy agreements or collaborative practice agreements allow the provision of test-to-treat services. By providing test-to-treat services, pharmacists can increase access to care for rural patients and alleviate the burden of offering these services from other healthcare providers.

## 1. Introduction

A key social determinant of health, as noted by Healthy People 2030, is access to healthcare [1]. In the United States, around 60 million people, or about one in five Americans, live in rural locations [2]. Rural populations often face challenges reaching healthcare facilities and services due to a variety of barriers, including personal barriers, such as finances, transportation, literacy, and trust; barriers to services include lack of clinics or providers, limitations to insurance coverage, stigma of receiving healthcare services, and distance to receiving healthcare services [3].

In rural areas, the closure rate of healthcare facilities is impacting the provision of services to residents who live there. There is a challenge in rural areas to provide healthcare services, as these areas frequently rely on small, critical access hospitals and hospital-affiliated clinics or stand-alone, independent clinics. The closure of healthcare facilities in the past 20 years has accelerated, often due to factors such as difficulty hiring and maintaining providers and ancillary support staff and a decrease in patient volume, complexity of health conditions treated, increasing costs, low reimbursement rates, and regulatory barriers [4,5,6,7]. The closure and loss of these facilities result in “medical deserts”, geographic regions possessing significant barriers in accessing healthcare, including having to travel more than an hour to obtain healthcare services [6,7,8,9,10].

However, even in rural areas and isolated communities where there is a loss of critical access hospitals and clinics, community pharmacies frequently continue to thrive. As such, many rural communities depend on community pharmacies to fill the gap in care. One way to address a lack of services usually provided by traditional clinic-based services is to enhance the availability of basic medical care in programs offered in community pharmacies [11,12,13,14,15]. Many rural community residents depend on local pharmacists and pharmacies to provide basic care and medical needs [16,17,18,19,20]. Expanding access to healthcare, through specialized services offered in rural pharmacies, can enhance and improve care to rural populations. While telepharmacy services have advanced in recent years, especially after the coronavirus disease 2019 (COVID-19) pandemic, patients often have needs that require in-person assessment [21,22,23,24,25,26,27,28,29,30].

In many states, expanded access to care can be obtained in pharmacies through test-to-treat programs, which typically involve point-of-care testing (POCT), utilizing a clinical lab test at a site to provide a quick turn-around of a diagnostic [31]. Test-to-treat programs often incorporate collaborative drug therapy agreements (CDTAs), also known as collaborative practice agreements (CPAs). CDTAs and CPAs are pre-prepared, specific written guidelines or protocols that authorize a pharmacist to specifically prescribe for a patient in a specific setting [32]. Typical examples include rapid strep testing, influenza, COVID-19, urinary tract infections, HIV, and immunization protocols [12,33]. This process is known as test-to-treat, since pharmacists can perform the test and prescribe treatment if the test is positive and the patient meets specific criteria. Offering test-to-treat services allows pharmacists to enhance access to care services for patients as extenders of care for primary care providers, including physicians, nurse practitioners, dentists, and other healthcare providers [34]. Expansion of care for conditions that are determined by a positive or negative test allows for expedited care of the patient and access to care when it otherwise may be limited [12,18,34]. This is especially important in rural areas, where clinic hours and availability of a provider may be limited or not available at all [35]. In these cases, test-to-treat services may be the difference in obtaining timely care for an acute condition.

In Washington State, pharmacists have had prescriptive authority with CDTAs since 1991 [36,37,38]. This permits a pharmacist, via an approved and filed CDTA at the state board of health with any practitioner licensed in the State of Washington, to prescribe legend drugs [36,37,38].This has had broad-reaching impacts in settings from hospital systems for initiation, dosing, and adjustment protocols for drug management and renally eliminated drug dose adjustments, to anticoagulation clinic management, to ambulatory care settings managing lipids, seizures, and HIV treatment, and to community-based pharmacies providing vaccine clinics, opioid overdose management, and communicable disease diagnosis and management [33,39,40,41,42,43,44,45]. These wide-reaching programs have expanded access to care to vulnerable populations, especially those residing in rural and underserved areas. However, recent national commentaries raised the question of infringement on the scope of practice rather than collaborative care of at-risk patients [46]. The objective of this qualitative study was to describe the benefits of and barriers to the provision of test-to-treat services for influenza, strep throat, and COVID-19 delivered by pharmacists. This objective was accomplished through the analysis of the results of key informant interviews of registered pharmacists in the State of Washington.

## 2. Materials and Methods

### 2.1. Theoretical Framework

The framework utilized for this qualitative research was Penchansky and Thomas’s “5 A’s of Access” [47]. This framework considers five domains that are critical for feasibility and implementation of healthcare services and the expectations both providers and clients have of said services. The domains are affordability, which includes the financial aspects of accessing care; availability, referring to whether or not required resources are available; accessibility, which describes the ease with which patients can obtain care; accommodation, describing the ability of providers’ operations to meet patient expectations and needs; and acceptability, which includes the patient and other professionals’ confidence in the provider’s skills [47]. This framework has been used to evaluate many aspects of healthcare access, including the COVID-19 pandemic impact on antiviral therapy [48] and children’s access and barriers to preventative care [49]. The framework was applied to our findings due to prominent themes that arose from the individual interviews, which primarily addressed the feasibility and implementation of test-to-treat protocols described by the study participants.

### 2.2. Study Design

A qualitative, descriptive study design was utilized to conduct in-depth, semi-structured key informant interviews with practicing pharmacists. Study methods were designed using the Consolidated Criteria for Reporting Qualitative Research (COREQ) guide [50] and principles of qualitative research [51,52,53] to ensure rigor in data collection and analysis methodology. The Institutional Review Board at Washington State University was consulted and found these research methods to meet criteria for research exempt from a full board review (IRB# 20146).

### 2.3. Interview Participants

One hundred sixty-seven potential participants were identified from a list of university-affiliated experiential preceptors with active community and ambulatory care rotation sites in Washington State. This list was provided by the experiential services team at the College of Pharmacy and Pharmaceutical Sciences at Washington State University. Potential participants were contacted individually via email by the primary investigator (B.B.). The recruitment email’s utilized language was reviewed and approved by the WSU IRB. These individuals were informed the interview would take approximately 20 min, participation would be voluntary, and they were free to skip individual questions or end the interview at any time. Interviews would be transcribed and analyzed in aggregate, with individually identifiable information removed. Potential participants were asked to respond directly to the recruitment email to identify a meeting time and to ask questions about the study as needed. Interviews were scheduled at a time convenient for the participant and interviewer. Individuals who did not respond were not contacted a second time.

### 2.4. Script Development

A 14-question semi-structured interview script was developed initially by the primary investigator (B.B.) with guidance from a faculty mentor (M.U.). The semi-structured interview format allowed the research to utilize a script while also including probing questions where needed to elicit in-depth responses [51,52]. The initial draft was shared and reviewed by a pharmacist researcher (K.M.). Feedback about script content and flow was provided and incorporated. After the script was finalized, it was piloted with another practicing pharmacist with experience conducting key informant interviews (A.S.). The interview script is shown in Appendix A.

### 2.5. Data Collection

Interviews were conducted via Zoom by one researcher (B.B.). The interviewer was a PharmD student who had been trained to conduct key informant interviews by faculty with qualitative research experience (K.M.). The interviewer had not directly interacted with any of the participants prior to conducting the interviews. The participant’s consent to have the interview recorded was confirmed at the beginning of the conversation. Audio files were transcribed using the Zoom transcription function. The primary investigator individually reviewed each transcript and corrected errors using the interview audio files. He also removed identifying information and assigned each participant a letter (Pharmacist A, Pharmacist B, etc.) before sharing the transcripts with another researcher for analysis.

### 2.6. Data Analysis

Two researchers met to discuss thematic coding procedures as described by Tolley et al. [51]. Researchers conducted first-level coding of one transcript together (B.B. and K.M). In first-level coding, meaningful phrases were identified, and individual quotes were organized using the constructs of the theoretical framework [51]. A codebook, a spreadsheet used to organize the phrases and quotes within the domains of the theoretical framework, was developed using Microsoft Excel. Second-level coding was conducted by two researchers via Zoom to organize the meaningful phrases into themes organized by the domains of the A’s of Access framework. The COREQ checklist was reviewed again to ensure details required for reporting standards were followed [50].

## 3. Results

Twenty key informant interviews were conducted between January and June in 2024. Thirteen unique counties out of 39 total in Washington State were represented by the pharmacists interviewed. Participant demographics are shown in Table 1.

Qualitative analysis elicited the following themes organized by the domains of the theoretical framework.

### 3.1. Accessibility

Accessibility refers to geographic accessibility, “which is determined by how easily the client can physically reach the provider’s location” [47]. Utilizing test-to-treat offered by pharmacies can improve patients’ access to care through decreasing the distance patients must travel to access treatment.

#### Theme 1: Pharmacists Can Reduce Geographical Barriers to Care by Offering Test-to-Treat Services

- “I think [pharmacists] definitely fill a gap that is much needed…some folks have barriers to care at their physicians’ offices whether that’s travel, whether it’s cost. And ultimately the pharmacists are the most readily available healthcare provider in the community”. Pharmacist F.
- “I think [Test-to-Treat] is awesome, especially in the rural communities. Let me give you an example. So let’s take [name of town], Washington population around 2000; small community, limited access to healthcare, which there are a lot of in Washington State. It is a great opportunity to be able to serve the community and their patients so they don’t have to travel 60 plus miles just to find an urgent care, or an emergency room, or a physician”. Pharmacist G.

### 3.2. Acceptability

Acceptability “captures the extent to which the client is comfortable with the more immutable characteristics of the provider, and vice versa” [47]. In this case, are test-to-treat services offered by pharmacists acceptable to patients? Pharmacists interviewed noted that in many cases, patients were either not aware that pharmacies offer test-to-treat services or that patients were so used to going to urgent care for all their medical needs that it did not occur to them that they could receive these services in a pharmacy. Pharmacists identified a lack of outreach to the public about what pharmacists are trained to do and education about services they are capable of providing.

#### 3.2.1. Theme 2. Patients Are Often Unaware That Test-to-Treat Services Are Available in Pharmacies

- “I think one of the challenges is the community’s understanding that pharmacists are qualified to do this. And there’s definitely a perception that people need to see their doctor for things”. Pharmacist B.
- “People don’t automatically think, ‘Oh, I can go to the pharmacy to get a rapid test for COVID-19, flu or strep’ they think, ‘Oh, I need to go to urgent care, or emergency care’…Some people don’t want to go to urgent care because of time to wait. And again, getting hold of their primary can be a little difficult, so I think knowledge is probably the biggest problem”. Pharmacist I.

In addition to the acceptability of test-to-treat services to patients, interview participants also remarked on the willingness of other healthcare providers to support these services. Just as pharmacists reported experiencing confusion from patients that pharmacies offer test-to-treat services, unfamiliarity was also present among other healthcare professionals.

#### 3.2.2. Theme 3. When Offering Test-to-Treat Services, Engaging in a Positive Relationship with Other Local Healthcare Providers Can Overcome Initial Skepticism

- “It’s funny. I’ve been around long enough that I was one of the first pharmacists to become an immunizer in the State of Washington, and there was quite a bit of pushback from doctors at that point. They were wondering why pharmacists were trying to muddle in their business. And it’s funny because now people go to their physician [for an immunization] and their physician refers them to the pharmacist. It just shows how the trends have changed over the years. So, we can test and treat”. Pharmacist S.
- “It can be difficult as a community pharmacist [discussing COVID-19 treatment] with primary care because you don’t know them. You don’t have a relationship with them, and I think that is one of the biggest barriers. Even with clinical knowledge and that training, if they don’t know you it is going to be hard for them to trust you”. Pharmacist E.
- “You know, anytime you’re introducing any kind of overlap in service from providers and pharmacists I think there is some initial skepticism. Within [my healthcare system] pharmacists have been embedded in collaborative practice agreements and just collaborative care for a very long time, so the culture where I work is very positive… I still think, even from that aspect, providers would generally view this as a positive thing. I think when you get into maybe some slightly more complex diseases, they are a little bit more standoffish, but I truly think that we’re still headed in a positive direction”. Pharmacist G.

### 3.3. Availability

Availability “measures the extent to which the provider has the requisite resources, such as personnel and technology, to meet the needs of the client” [47]. The availability construct considers the needs of the patient and whether providers can meet those needs. Many subjects described the lack of healthcare options in the areas they work and how pharmacies could address this need. Not only would pharmacists providing these services save time for patients, but it could also help lessen the heavy caseload burden for other providers.

#### 3.3.1. Theme 4: By Offering Test-to-Treat Services, Pharmacies Can Reduce the Wait Time for Patients Seeking Care

- “I think it’s really important for pharmacists to provide [Test-to-Treat]. It’s a great service that prevents emergency room visits. I’m in a very rural community, and there are no walk-in clinics”. Pharmacist B.
- “Quick! 15 min in and out. Pharmacies now have adapted to have testing areas or rooms that came with immunizations… we allow people to get in a lot faster than their providers at this point”. Pharmacist T.

#### 3.3.2. Theme 5: By Offering Test-to-Treat Services, Pharmacies Can Reduce the Number of Rural Patients Seeking Higher Levels of Care for Basic Treatments

- “I see it as a way of better utilizing not only [pharmacists’] skills but utilizing the healthcare system as a whole. Waiting time for ER’s are always crazy. To wait that long just to get a strep test or a COVID-19 test or a flu test seems kind of silly to me if it’s something that can be done at your community pharmacy. Not only are we saving those providers at the urgent cares and emergency departments’ time, we’re also saving the patients’ time”. Pharmacist U.
- “I think [Test-to-Treat] shifts the need for more simple diagnoses over to pharmacists, and that generally frees up scheduling and availability for nurse practitioners, physician assistants, and MDs to be able to diagnose maybe more complex disease states than pharmacists would be able to. So, it just opens up lines of access and care”. Pharmacist G.

In some cases, pharmacists reported that local physicians saw the value in this reduction in workload and supported the pharmacist in offering these services:

- “I think that most doctors have a high regard for pharmacists and as long as they know what type of training the pharmacists have undergone. I think the majority of them are willing to allow pharmacists to go ahead and expand the scope of practice that we as pharmacists have”. Pharmacist S.

In addition to considering the availability of services for the patient, this construct can also include resources needed for offering this service, including staffing, supplies, and training.

### 3.4. Accommodation

Accommodation “reflects the extent to which the provider’s operation is organized in ways that meet the constraints and preferences of the client” [47]. This domain reflects some of the primary difficulties for the pharmacies themselves involving challenges of the implementation of test-to-treat, including the logistics of service. While there has been a large focus on the benefits that would arise from pharmacists being enabled to test-to-treat at a community level, for many pharmacies, it would be difficult to integrate a new service when they have full hands with their current responsibilities.

#### Theme 6: Offering Test-to-Treat Services Can Be Beneficial to Rural Communities but Poses Challenges for Rural Pharmacies

- “I can tell you it’s I think it’s a good thing for the community, but not necessarily for the pharmacy team. The reason I say that is because [Test-to-Treat] just adds to their workflow. And they already have a lot on their plate with giving vaccines and filling prescriptions and answering phone calls”. Pharmacist O.
- “[Test-to-Treat] barriers during COVID-19 were staff allocation and workflow balancing. Trying to address all the needs of the pandemic while trying to continue address the needs of our pharmacy patients. That was a little bit of a challenge for us, specifically”. Pharmacist Q.

While there are advantages for the community and patient populations, there would be a cost or adjustment for the pharmacies engaging in test-to-treat protocols. Similarly, pharmacies need to have the capacity to build in test-to-treat into the pharmacy workflow. Pharmacy tasks related to dispensing prescriptions would still need to be offered simultaneously. Pharmacists described the need to ensure adequate staffing:

- “[Offering Test-to-Treat services] shouldn’t be stopping any of the regular stuff that we do. It should just be able to go into the workflow and not prevent us from completing all of our tasks throughout the day”. Pharmacist D.
- “I think the biggest challenge would be being adequately staffed in order to meet the need to provide these services… because pharmacists are already expected to do a lot of things with very little staff”. Pharmacist K.
- “I think the largest stumbling block for pharmacists is time…time is a huge asset that pharmacists have, and they have to use their time wisely. I just want to make sure that pharmacists are provided adequate time to do these types of encounters with patients”. Pharmacist S.

### 3.5. Affordability

The affordability domain of the framework is “determined by how the provider’s charges relate to the client’s ability and willingness to pay for services” [47]. In this context, affordability involves considering the willingness of patients or insurance companies to pay for test-to-treat services provided by pharmacists. The main theme identified in this domain was difficulties with billing or reimbursement issues that the pharmacist encountered, which hindered service sustainability.

#### Theme 7: Pharmacists Experienced Difficulties with Receiving Sufficient Payment for Test-to-Treat Services to Offset Costs

Many subjects expressed their concerns regarding insurance coverage and whether reimbursement for services would cover the costs of providing testing, including staff time and testing supplies:

- “I really think just billing is maybe the main hurdle of having insurances be willing to cover our services”. Pharmacist G.
- “The time to bill as well as navigating that process and ensuring reimbursement [is difficult]. The rate of reimbursement compared to the time to train pharmacists for these services, get the supplies, organize the supplies, administer the test, get the treatment ready… is not equitable to the amount of time that pharmacists and support staff are providing for this”. Pharmacist J.

## 4. Discussion

This study sought to identify and describe the perceptions of practicing pharmacists regarding the benefits of and barriers to providing test-to-treat services at their patient care sites. By applying the 5 A’s of Access framework to evaluate responses from key informant interviews with pharmacists, detailed descriptions of these barriers and benefits were derived. When determining the value of pharmacy test-to-treat services, the initial focus must be on whether these services are valuable to the patient. An estimated 90% of the USA population lives within five miles of a pharmacy [54], and pharmacies are often more accessible than medical clinics [54,55], even in rural communities. For some patients, there may be a hesitancy to seek treatment for minor ailments and conditions because of the perception that these issues can only be treated by a physician, but there are barriers to receiving treatment, such as long wait times and traveling long distances. Pharmacists in the study identified that offering test-to-treat services provided benefits in their communities, such as increasing access to these services for patients and reducing the geographical distance that patients must travel to access care. Costs and time associated with healthcare-related travel can be a substantial burden for patients in rural communities, particularly those with limited resources. Rural communities often lack public transportation, which can be a barrier to care [56]. Results showed that pharmacists believed offering test-to-treat services could increase the availability of care, particularly in our rural communities, where care is sometimes lacking.

In addition to directly increasing access to care for patients, pharmacy test-to-treat services provide an additional benefit of reducing the workload burden for other rural healthcare providers. Interview respondents recognized that providing test-to-treat services has the potential to positively impact the health system by offloading the management of minor respiratory ailments from other members of the care team and reducing long wait times in clinics, urgent care centers, and emergency rooms. This could indirectly increase the availability of patient care services in their rural communities by allowing physicians to manage more serious conditions. As rural areas in the United States face unprecedented care shortages [57], empowering pharmacists to offer test-to-treat services could be a valuable tool to help meet care demand.

In addition to increasing access, it is essential that offering test-to-treat services in pharmacies does not compromise the quality of existing care or lead to additional costs incurred by the patient. In a recent commentary by Smith and Colleagues, barriers to the full implementation of pharmacist-provided test-to-treat services were identified as related to socio-political constructs, resource allocation, and competence [58]. However, a recent publication by Akers and colleagues [12] demonstrated that pharmacist-provided care for minor ailments and conditions, including test-to-treat services, was equally as effective as care provided in more traditional settings, including primary care, urgent care, and the emergency department. Cost of care must also be considered. In addition to confirming that care services offered by pharmacists were equivalent in quality, results of the study also showed that it was less costly than care provided in traditional care setting [12].

It is positive to note that our survey respondents did not identify competence, education, or training as significant barriers to the implementation of test-to-treat services. Although pharmacists and their staff must be adequately trained to provide safe and effective patient care services, our results may indicate that pharmacists are receiving adequate training in their Doctor of Pharmacy curricula and have access to relevant professional development resources through their state and national associations. The Accreditation Council for Pharmacy Education (ACPE) recently approved updated standards for schools and colleges of pharmacy that go into effect in July 2025 [59]. In addition to highlighting the expectation that curricula prepare students for conducting thorough patient assessment, including laboratory data interpretation, the new standards specifically call out prescribing as a new skill to be taught to pharmacy students. This update is consistent with and will support the expansion of pharmacist-provided patient care, including test-to-treat services, as graduates around the country enter the workforce with these necessary skills and the expectation that they will be utilized as part of their practice.

Key challenges to offering test-to-treat services identified by study pharmacists were general skepticism and a lack of support from other rural healthcare providers. These results align with a recent commentary against pharmacist expansion of services from the American Medical Association [46]. Smith and colleagues also described challenges related to the acceptance and confidence of traditional healthcare providers as a barrier to overcome [Smith]. Interview participants emphasized the importance of building trusting relationships with physicians, which aligns with similar results from a 2019 study evaluating the role of the pharmacist in managing chronic conditions in rural communities [60]. Ideally, pharmacists looking to implement test-to-treat services have well-developed professional relationships with the other providers in their settings and can leverage those relationships to increase support from other providers. One interview participant compared physician apprehension and pushback related to offering test-to-treat services in pharmacies with similar feelings about offering vaccination services in the early 2000s. Pharmacies now offer more vaccinations than any other provider type [61].

Interview respondents expressed concerns about maintaining existing pharmacy dispensing services when new services are implemented. Many pharmacies, especially those in rural areas, struggle to ensure adequate staffing to meet regulatory requirements and to maintain traditional services. They may be unable to increase staffing, even if new services have the potential for community benefit and income. Another cost-related barrier described by study pharmacists was reimbursement for services. Challenges with reimbursement for test-to-treat services pose a significant threat to growth in access to this care. Smith and colleagues also described potential challenges related to the supply chain for needed supplies and medications, as well as gaps in technology necessary for reporting, surveillance, and documentation. Our study participants did not highlight these potential barriers in their responses, but addressing inventory and electronic health record access is critical to the success of test-to-treat services. Study respondents almost uniformly expressed concerns about the challenges in receiving adequate third-party reimbursement for delivering these new clinical services. Akers and colleagues identified that test-to-treat services in pharmacies cost less for third-party payors and patients alike, compared to the equivalent services offered in traditional care sites [12]. To increase access to pharmacist-provided test-to-treat services, additional training, support, and streamlined processes for contracting and billing insurance will be necessary.

Lastly, pharmacists described a lack of awareness about available test-to-treat services among the public as a barrier to successful delivery. The public is often unaware that pharmacists are trained to provide direct patient care services, such as test-to-treat. This lack of understanding may lead patients to go directly to clinics, emergency departments, or urgent care centers to seek this type of care. Future education and marketing campaigns for the public and for other healthcare providers are needed before these pharmacist-provided services may be of value.

This study has several limitations to note. Key informant interviews asked participants to recall experiences and provide opinions, which can introduce recall bias and recency bias. Interview participants in this study practice in Washington State, where pharmacists are routinely authorized to assess patients and prescribe medications via a collaborative drug therapy agreement, so it is not surprising that concerns for legal and regulatory barriers were not highlighted. The results of this study may not be representative of pharmacists in states where they do not routinely prescribe medication or assess patients because of practice act limitations.

The key informant interviews were also conducted by a student pharmacist (B.B.) who was mentored by faculty researchers. Having only one interviewer enabled consistency in the data collection process but may also have introduced limitations. Due to limited pharmacy practice experience, the student interviewer may not have recognized all opportunities to probe respondents for additional relevant details in their responses. Study respondents were also preceptors for WSU. It is possible that these preceptors described a more positive practice environment than existed because the interviewer was a student.

The strengths of this project include the qualitative research methodology that was employed and the variety of respondents that contributed to the results. Additionally, there was no external funding for this project, so there was no potential for undue, outside influence. Although small in number, respondents came from a variety of pharmacy practice sites and geographies across the state, which increases confidence in the completeness and generalizability of the results.

## 5. Conclusions

Rural communities experience challenges with the limited capacity of healthcare providers to meet the needs of patients in their communities. The results of this qualitative analysis may be useful to pharmacists in U.S. states where CDTAs or CPAs allow the provision of test-to-treat services. By providing test-to-treat services, pharmacists can increase access to care for rural patients and alleviate the burden of offering these services from other healthcare providers. Pharmacists offering test-to-treat services face challenges regarding receiving sustainable reimbursement for services. Education and marketing materials may also be needed to improve rural patient knowledge of available services. Finally, efforts are needed to support and expand the pharmacy workforce, particularly in rural communities, to ensure staffing levels are adequate for the maintenance of necessary dispensing services but also allow for growth in patient care services.

## Figures and Tables

**Table 1:** Interview participant demographics.

Demographic	N (%)
Practice site	
Ambulatory Care	7 (35%)
Chain Community Pharmacy	6 (30%)
Independent Community Pharmacy	5 (25%)
Home Health Consulting	1 (5%)
Specialty	1 (5%)
Years working as a pharmacist	
Average	15.4 years
Range	3–39 years
Years working at practice site	
Average	7.1 years
Range	1–25 years

## Data Availability

The data presented in this study are available on request from the corresponding author.

## Abbreviations

The following abbreviations are used in this manuscript:

POCT	Point-of-Care Testing
CPA	Collaborative Practice Agreement
CDTA	Collaborative Drug Therapy Agreement
COVID-19	Coronavirus Disease 2019

## Appendix A

**Table A1:** Key informant interview script.

Key Informant Interview Script
How many years have you been a practicing pharmacist?
2. How many years have you been at your current practice site?
3. Which of the following best describes your practice site: **a.** Independent community pharmacy **b.** Chain community pharmacy **c.** Ambulatory care/clinic pharmacy **d.** Institutional pharmacy **e.** Consulting pharmacy **f.** Specialty pharmacy **g.** Other (please describe)
4. Which county in Washington State do you practice in?
5. What training do you have to offer test-to-treat for COVID-19, flu, and strep throat?
6. What is your current scope of practice regarding COVID-19, flu, and strep throat? (“Current scope of practice” is defined as the activities that an individual healthcare practitioner is permitted to perform within their specific profession).
7. If you provide rapid testing and treatment, otherwise referred to as “test-to-treat”, for COVID-19, flu, and strep throat, how does it benefit other healthcare providers?
8. If you provide rapid testing and treatment, otherwise referred to as “test-to-treat”, for COVID-19, flu, and strep throat, how does it not benefit other healthcare providers?
9. Should a pharmacist perform test-to-treat for COVID-19, influenza, and streptococcal pharyngitis?
10. Why or why not?
11. If a patient is suspected of having COVID-19, flu, or strep throat at your practice site, what procedures are currently in place for diagnosis and treatment of the patient?
12. As a pharmacist in a rural area, how would “test-to-treat” for COVID-19, flu, or strep benefit your specific patient population?
13. As a pharmacist in a rural area, how would “test-to-treat” for COVID-19, flu, or strep benefit or how would it not benefit your specific patient population?
14. If pharmacists receive proper training and are enabled to provide rapid testing and treatment for COVID-19, flu, or strep throat, will the United States healthcare system be better prepared to handle future outbreaks?
